# vNN Web Server for ADMET Predictions

**DOI:** 10.3389/fphar.2017.00889

**Published:** 2017-12-04

**Authors:** Patric Schyman, Ruifeng Liu, Valmik Desai, Anders Wallqvist

**Affiliations:** DoD Biotechnology High Performance Computing Software Applications Institute, Telemedicine and Advanced Technology Research Center, U.S. Army Medical Research and Materiel Command, Fort Detrick, MD, United States

**Keywords:** ADME, toxicology, QSAR, machine learning, applicability domain, online web platform, open access

## Abstract

In drug development, early assessments of pharmacokinetic and toxic properties are important stepping stones to avoid costly and unnecessary failures. Considerable progress has recently been made in the development of computer-based (*in silico*) models to estimate such properties. Nonetheless, such models can be further improved in terms of their ability to make predictions more rapidly, easily, and with greater reliability. To address this issue, we have used our vNN method to develop 15 absorption, distribution, metabolism, excretion, and toxicity (ADMET) prediction models. These models quickly assess some of the most important properties of potential drug candidates, including their cytotoxicity, mutagenicity, cardiotoxicity, drug-drug interactions, microsomal stability, and likelihood of causing drug-induced liver injury. Here we summarize the ability of each of these models to predict such properties and discuss their overall performance. All of these ADMET models are publically available on our website (https://vnnadmet.bhsai.org/), which also offers the capability of using the vNN method to customize and build new models.

## Introduction

Drug discovery is a risky, lengthy, and resource-intensive process with high attrition rates. In recent years, the development of assays and computer-based (*in silico*) models to assess absorption, distribution, metabolism, and excretion (ADME) properties has greatly reduced the attrition rate (Waring et al., 2015). The ability to predict these properties quickly and reliably facilitates the exclusion of compounds with potential ADME issues, and thereby helps investigators prioritize which compounds to synthesize and evaluate. However, toxicity remains a hurdle, with an attrition rate of 40% among new compounds identified in the drug discovery phase (Waring et al., 2015). This necessitates careful selection of compounds during drug development to avoid late-stage attrition. As such, there is an urgent need for *in silico* methods that make fast, easy, and reliable predictions of ADME and toxicity (ADMET) properties, which has resulted in several online tools and web-platforms for ADMET predictions (Walker et al., 2010; Sushko et al., 2011; Cheng et al., 2012; Maunz et al., 2013; Manganaro et al., 2016; Daina et al., 2017).

Here we provide an overview of our versatile variable nearest neighbor (vNN) method (Liu et al., 2012) and the 15 models we constructed using this method to predict the ADMET properties of potential target compounds. The vNN method has several advantages over existing *in silico* methods. First, it calculates the similarity distance between molecules in terms of their structure, and uses a distance threshold to define a domain of applicability (i.e., all nearest neighbors that meet a minimum similarity threshold constraint). This applicability domain, while limiting vNN-based models to making predictions only for molecules that are similar to the reference molecules, ensures that the predictions they generate are reliable. Second, vNN-based models can be built within minutes and require no re-training when new assay information becomes available—an important feature when keeping quantitative structure—activity relationship (QSAR) models up-to-date to maintain their performance levels. Finally, as we show throughout this work, the performance characteristics of our vNN-based models are comparable, and often superior, to those of other more elaborate model constructs.

We have developed a publically available vNN website (https://vnnadmet.bhsai.org/). This website provides users with ADMET prediction models that we have developed, as well as a platform for using their own experimental data to update these models or build new ones from scratch. Although we use the vNN method here for predicting ADMET properties, the vNN website can be used to build a variety of classification or regression models.

## Materials and methods

### The vNN method

The k-nearest neighbor (k-NN) method is widely used to develop QSAR models (Zheng and Tropsha, 2000). This method rests on the premise that compounds with similar structures have similar activities. The simplest form of the k-NN method takes the average property values of the k nearest neighbors as the predicted value. However, because structurally similar compounds tend to show similar biological activity, it is reasonable to weight the contributions of neighbors so that closer neighbors contribute more to the predicted value. One notable feature of the k-NN method is that it always gives a prediction for a compound, based on a constant number, k, of nearest neighbors no matter how structurally dissimilar they are from the compound. An alternative approach is to use a predetermined similarity criterion. We developed the aforementioned vNN method, which uses all nearest neighbors that meet a structural similarity criterion to define the model's applicability domain (Liu et al., 2012, 2015; Liu and Wallqvist, 2014). When no nearest neighbor meets the criterion, the vNN method makes no prediction.

One of the most widely used measures of the similarity distance between two small molecules is the Tanimoto distance, *d*, which is defined as:

(1) $$d\;=\;1-\;\frac{n(P\cap Q)}{n\left(P\right)+n\left(Q\right)-n(P\cap Q)},$$

where *n*(*P* ∩ *Q*) is the number of features common to molecules *p* and *q*, and *n*(*P*) and *n*(*Q*) are the total numbers of features for molecules *p* and *q*, respectively. The features used to calculate molecular similarity are often based on atom type (connectivity and chemical properties), such as element, charge, donor, acceptor, and aromatic, but they can also be based on holistic molecular properties, such as molecular weight and partition coefficient (LogP). The predicted biological activity *y* is then given by a weighted average across structurally similar neighbors:

(2) $$y\;=\;\frac{\sum_{i\;=\;1}^{\nu }y_{i}e^{-{\left(\frac{d_{i}}{h}\right)}^{2}}}{\sum_{i\;=\;1}^{\nu }e^{-{\left(\frac{d_{i}}{h}\right)}^{2}}},\;d_{i}\;\leq \;d_{0}$$

where *d*_*i*_ denotes the Tanimoto distance between a query molecule for which a prediction is made and a molecule *i* of the training set; *y*_*i*_ is the experimentally measured activity of molecule *i*; *h* is a smoothing factor, which dampens the distance penalty; *d*_0_ is a Tanimoto-distance threshold, beyond which two molecules are no longer considered to be sufficiently similar to be included in the average; and *v* denotes the total number of molecules in the training set that satisfy the condition *d*_*i*_ ≤ *d*_0_. The values of *h* and *d*_0_ are determined from cross-validation studies.

To identify structurally similar compounds, we used Accelrys extended-connectivity fingerprints with a diameter of four chemical bonds (ECFP4) (Rogers and Hahn, 2010). For the vNN website, we chose ECFP4 fingerprints, which have previously been reported to show satisfactory overall performance in retrieving the active compounds of diverse datasets (Hert et al., 2004; Duan et al., 2010; Schyman et al., 2016). We emphasize that *h* and *d*_0_ are unique, and need to be optimized for each set of fingerprints and training set.

### Model validation

We used the 10-fold cross-validation (CV) procedure to validate the model and determine the values of *h* and *d*_0_. We randomly divided the data into 10 sets, 9 of which we used to develop the model and the 10th to validate the model. We repeated this process 10 times, leaving each set of molecules out once. In the next section, we report averages of the 10-fold CV as the performance measures.

### Performance measures

We used the following metrics to assess the quality of the classification models:

(3) $$\text{sensitivity}=\frac{\text{TP}}{\text{TP }+\text{ FN}}$$

(4) $$\text{specificity}=\frac{\text{TN}}{\text{FP }+\text{ TN}}$$

(5) $$\text{accuracy}=\frac{\text{TP }+\text{ TN}}{\text{TP }+\text{ TN }+\text{ FP }+\text{ FN}}$$

(6) $$\text{kappa}=\frac{\text{accuracy }-\;\mathrm{Pr}(\text{e})}{1\;-\;\mathrm{Pr}(\text{e})}$$

where TP, TN, FP, and FN denote the numbers of true positives, true negatives, false positives, and false negatives, respectively. The metric kappa assesses the quality of binary classifiers (Dunn and Everitt, 1995). Pr(*e*) is an estimate of the probability of a correct prediction by chance. It is calculated as:

(7) $$\mathrm{Pr}\left(\text{e}\right)=\frac{\left(\text{TP }+\text{ FN}\right)\left(\text{TP }+\text{ FP}\right)\;+\;(\text{FP }+\text{ TN})(\text{TN }+\text{ FN})}{{\left(\text{TP }+\text{ FN }+\text{ FP }+\text{ TN}\right)}^{2}}$$

The sensitivity measures a model's ability to correctly detect true positives, whereas the specificity measures its ability to detect true negatives. Kappa compares the probability of correct predictions to the probability of correct predictions by chance. Its value ranges from +1 (perfect agreement between model prediction and experiment) to −1 (complete disagreement), with 0 indicating no agreement beyond that expected by chance.

The performance measure for regression models is given by the Pearson's correlation coefficient (Adler and Parmryd, 2010):

(8) $$R\;=\frac{\sum_{i\;=\;1}^{n}\left(x_{i}-\bar{x})(y_{i}-\bar{y}\right)}{\sqrt{\sum_{i\;=\;1}^{n}{\left(x_{i}-\bar{x}\right)}^{2}}\sqrt{\sum_{i\;=\;1}^{n}{\left(y_{i}-\bar{y}\right)}^{2}}}$$

where *n* is the sample size, *x*_*i*_ and *y*_*i*_ are samples, and $\bar{x}$ and $\bar{y}$ are sample means. The correlation coefficient provides a measure of the interrelatedness of numeric properties. Its value ranges from −1 (highly anticorrelated) to +1 (highly correlated), and is 0 when uncorrelated.

We also calculated the coverage, which we define as the proportion of test molecules with at least one nearest neighbor that meets the similarity criterion. For all other molecules that do not meet the criterion, we do not make any predictions. In this case, the coverage is a measure of the size of the applicability domain of a prediction model.

## Results

### The vNN platform

**Figure 1:**
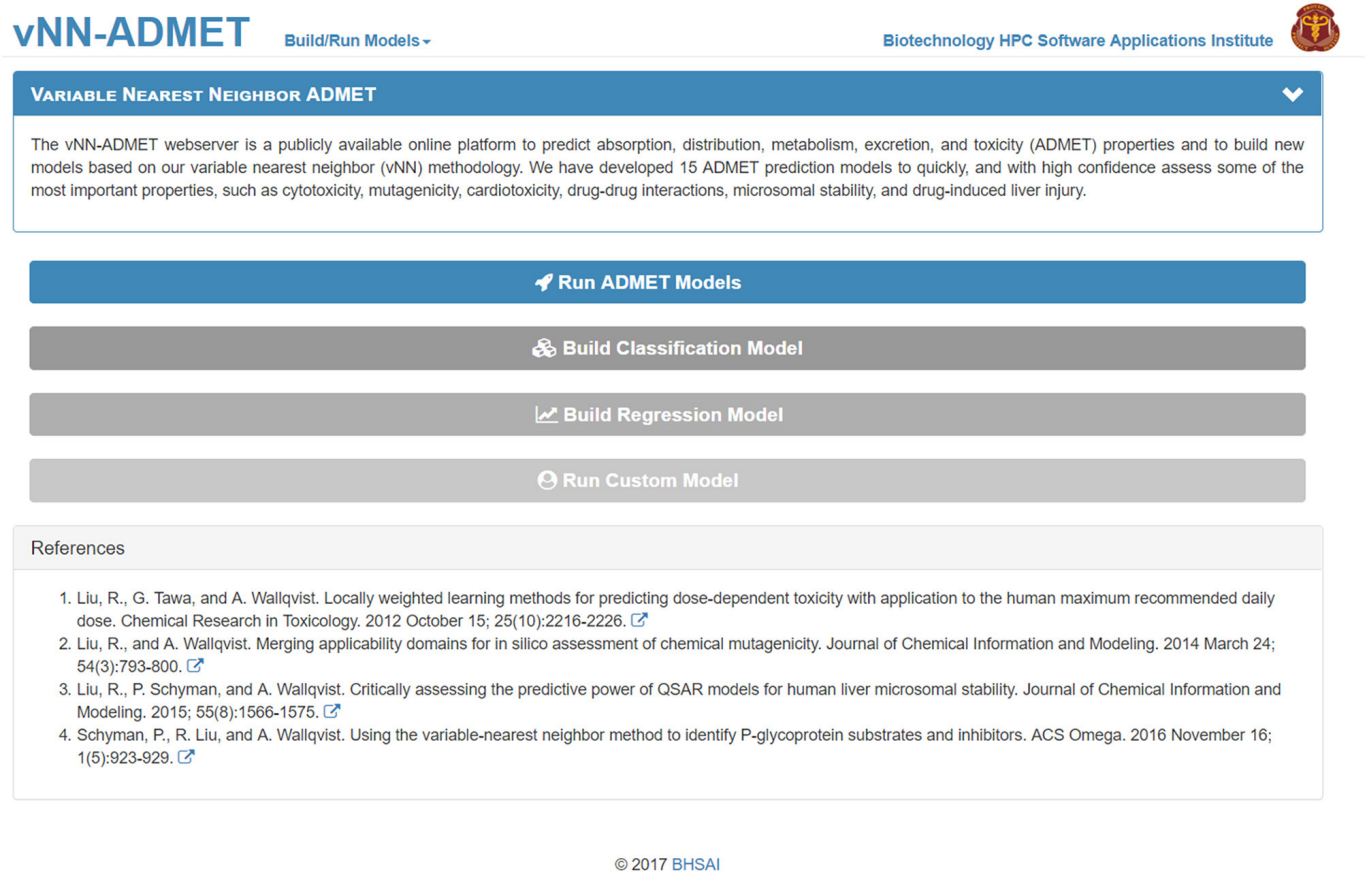
The vNN-ADMET main page. From this page, users can run ADMET models or build their own models.

The main purpose of the vNN-based platform is to provide users with a tool to make ADMET predictions and a user-friendly environment to build new models. Hence, the platform offers users two main capabilities that are accessible from the main webpage (https://vnnadmet.bhsai.org/) (Figure 1): (1) to run prebuilt ADMET models and (2) to build and run customized models.

**Figure 2:**
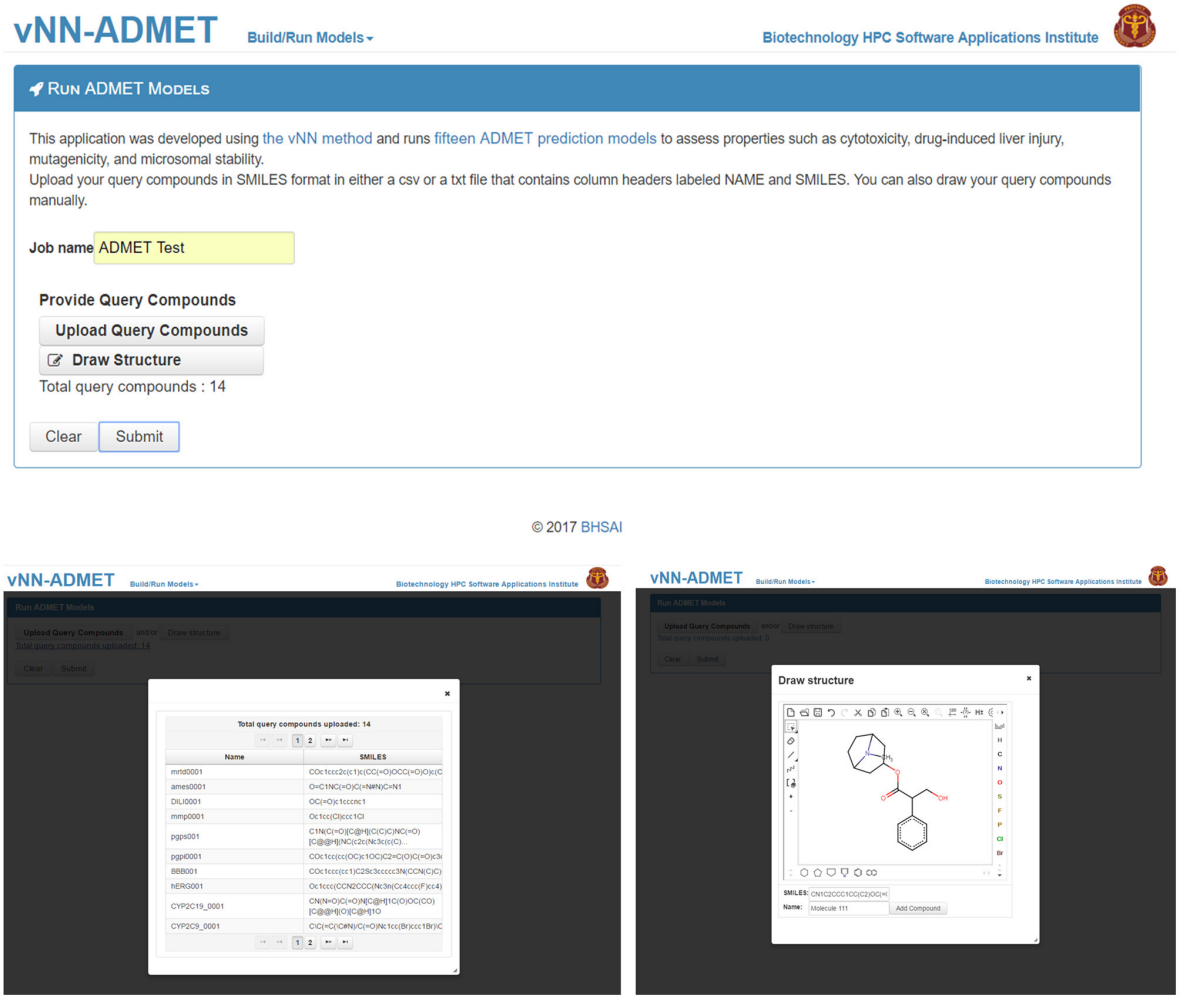
Submit ADMET predictions. On the *Run ADMET Models* page **(top)** users can upload a list of query compounds in SMILES format **(lower left)** or manually enter compounds by using the draw structure feature **(lower right)**.

**Figure 3:**
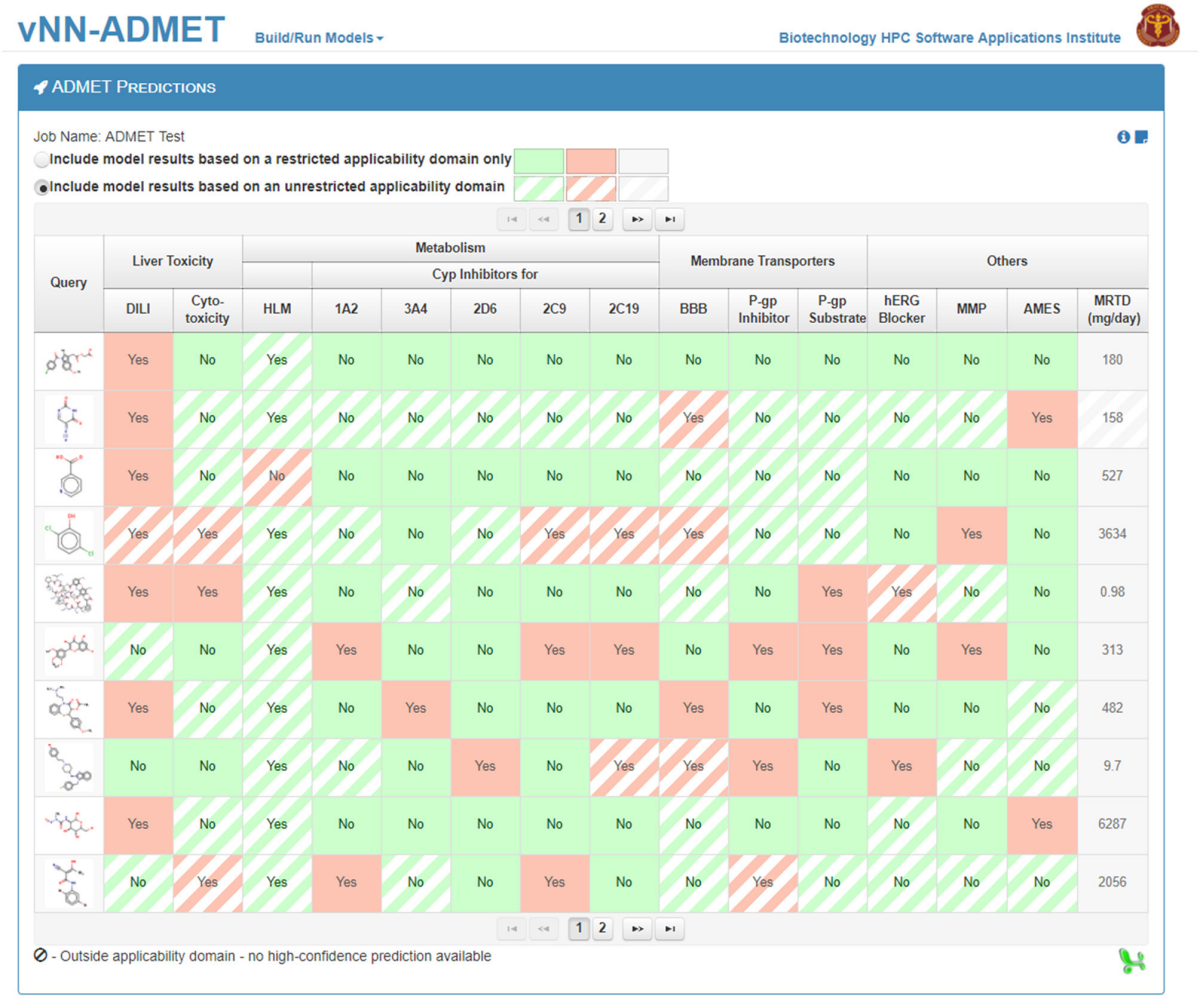
The ADMET predictions result page. The 15 ADMET predictions for each query molecule are presented on a separate row. Predictions based on models using a restricted applicability domain are shown in solid colors and those based on models using an unrestricted applicability domain are shown in striped colors. Users can download the results from the website into a single file.

To use prebuilt ADMET models, users need only provide one or more query molecules as the input (Figure 2). They can do this either by drawing the molecule, entering the molecular SMILES string (Weininger, 1988) directly on the website, or uploading a text file (csv or txt format) with query molecules in SMILES format. The text file should contain column headers labeled as NAME and SMILES. Once users upload the query molecules, they can submit the job. The application will then automatically run all ADMET prediction models. The output will be displayed once all predictions are completed and a temporary link to the result page will be sent to the user's e-mail address. The results can be downloaded as a table to the user's computer (Figure 3). By default, the user will see the ADMET results for our models, which use a restricted applicability domain. However, there is an option to include the results for the remaining compounds, using our unrestricted applicability domain models. The time required to run 100 query compounds is ~5 min on the server. However, this may vary depending on the size of the molecules and whether or not the job has been queued.

**Figure 4:**
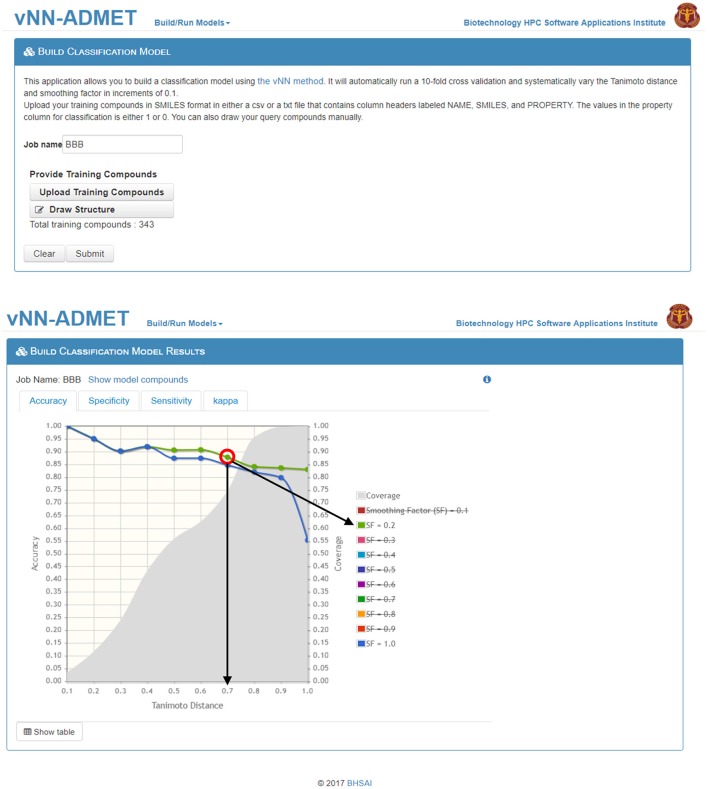
Build a classification model. On the *Build Classification Model* page **(top)**, users can upload their training data and/or draw structures. On the *Build Classification Model Results* page **(bottom)**, users can interactively select/deselect different smoothing factors for comparison. The graph shows accuracy of performance on the 10-fold cross validation test at different Tanimoto distances, where smoothing factors 0.2 and 1.0 are highlighted in green and blue, respectively (strikethrough smoothing factors indicate deselected values). The coverage is shown in gray. The red circle indicates the “best” model performance based on accuracy and coverage, where the black arrows show the corresponding Tanimoto-distance threshold (*d*_0_ = 0.7) and smoothing factor (*h* = 0.2). Although the accuracy is reduced to 88 from 90% at *d*_0_ = 0.6, the number of compounds predicted increases from 60 to 75%, which may be worth the loss in accuracy.

Users can build their own models by either selecting *Build Classification Model* or *Build Regression Model* on the main webpage (Figure 1). On the *Build Classification Model* page (Figure 4), users are asked to upload a list of molecules in SMILES format and the property of interest, with column headers labeled as NAME, SMILES, and PROPERTY. The value of the property should be set to 1 or 0 for classification models and real numbers for regression models. The vNN platform will then automatically run 10-fold CV by varying the Tanimoto distance (*d*) from 0.1 to 1.0 in increments of 0.1, and the smoothing factor (*h*) from 0.1 to 1.0 at each value of *d*. Once the calculations are completed, a temporary link to the result page will be sent to the user's e-mail address. The results will be displayed on an interactive webpage where users can select the values for *d* and *h* (Equation 2), depending on the optimal performance measures and coverage (Figure 4). The time required to build a model with a dataset of 1,000 compounds is ~10 min.

**Figure 5:**
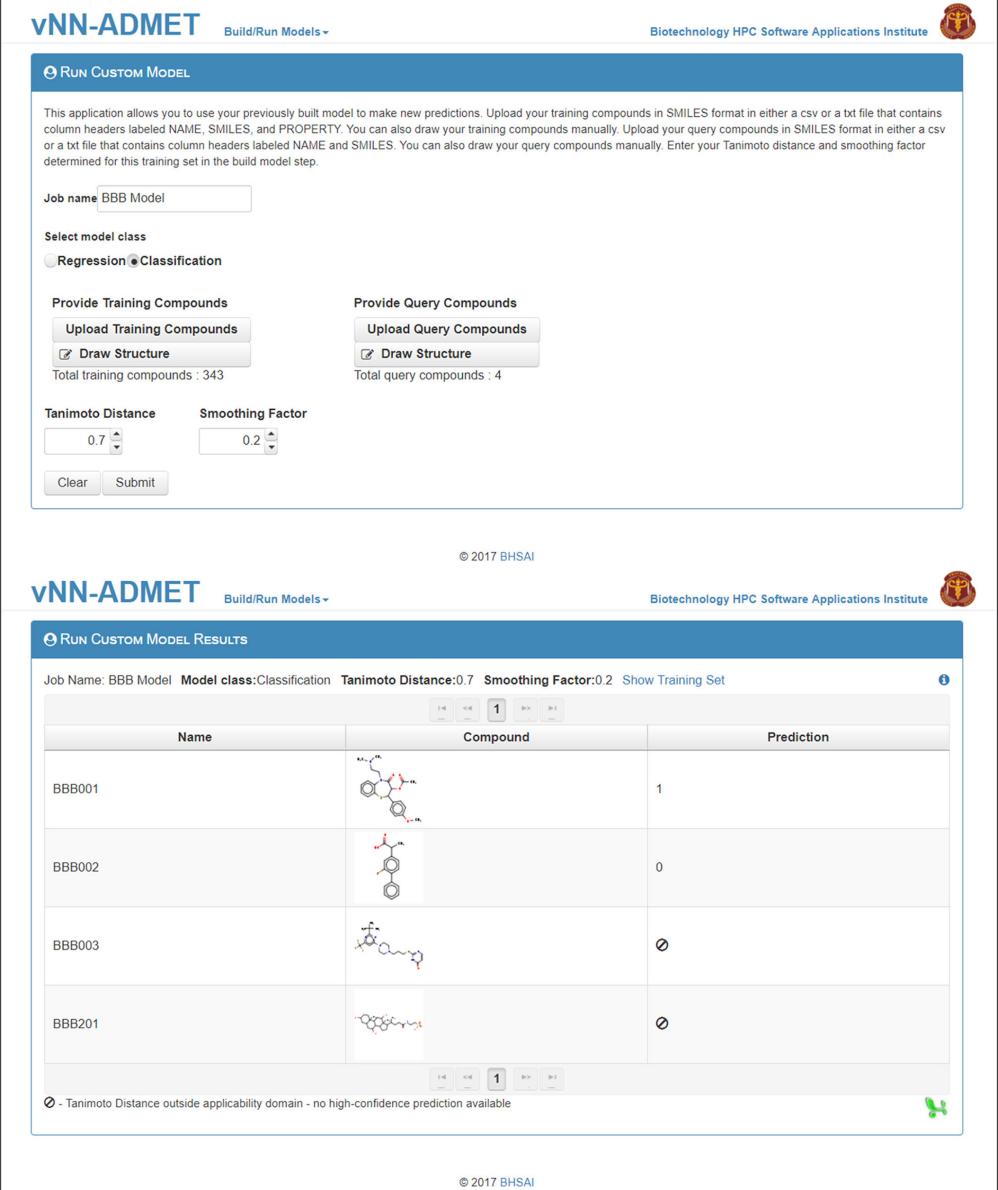
Run a customized model. The first step to run a customized model is to upload the training dataset, as well as the selected Tanimoto distance and smoothing factor from Figure 4. The second step is to upload query compounds. The results can be downloaded from the *Run Custom Model Results* page (bottom).

Users can then select the *Run Custom Model* option to predict the activity of new test molecules (Figure 5), using the previously selected values for the *Tanimoto Distance* and *Smoothing Factor*, and add the same molecules as those used to train the model in the *Upload Compounds with Property* data field. They then need to add the new query molecule(s) in SMILES format in the *Upload Query Compounds* field. The result will be displayed on a new webpage, and a temporary link to that page will also be sent to the user's e-mail address (Figure 5).

### Available ADMET predictions

The available ADMET prediction models, including their performance measures for the restricted applicability domain model, are summarized in Table 1. The performance measures for the models using an unrestricted applicability domain are presented in Table S1 in the Supplementary Material and on our website (https://vnnadmet.bhsai.org/). The 15 models cover a diverse set of ADMET endpoints. We will briefly describe these models and their performance measures, as well as the sources from which we retrieved the data. All datasets are available in SMILES format on the vNN web server or in Structure Data Format (SDF) in the Supplementary Material (Datasheet 1). Some of the models have already been published (Liu et al., 2012, 2015; Liu and Wallqvist, 2014; Schyman et al., 2016). We also present several new models here for the first time.

**Table 1:** Performance measures of vNN models in 10-fold cross validation, using a restricted applicability domain.

**Model**	**Data^a^**	***d*_*0*_^b^**	***h*^c^**	**Accuracy**	**Sensitivity**	**Specificity**	**Kappa**	***R*^d^**	**Coverage**
DILI	1,427	0.60	0.50	0.71	0.70	0.73	0.42		0.66
Cytotox (hep2g)	6,097	0.40	0.20	0.84	0.88	0.76	0.64		0.89
HLM	3,219	0.40	0.20	0.81	0.72	0.87	0.59		0.91
CYP 1A2	7,558	0.50	0.20	0.90	0.70	0.95	0.66		0.75
CYP 2C9	8,072	0.50	0.20	0.91	0.55	0.96	0.54		0.76
CYP 2C19	8,155	0.50	0.20	0.87	0.64	0.93	0.58		0.76
CYP 3A4	10,373	0.50	0.20	0.88	0.76	0.92	0.68		0.78
CYP 2D6	7,805	0.50	0.20	0.89	0.61	0.94	0.57		0.75
BBB	353	0.60	0.20	0.90	0.94	0.86	0.80		0.61
Pgp Substrate	822	0.60	0.20	0.79	0.80	0.79	0.58		0.66
Pgp Inhibitor	2,304	0.50	0.20	0.85	0.91	0.73	0.66		0.76
hERG	685	0.70	0.70	0.84	0.84	0.83	0.68		0.80
MMP	6,261	0.50	0.40	0.89	0.64	0.94	0.61		0.69
AMES	6,512	0.50	0.40	0.82	0.86	0.75	0.62		0.79
MRTD^e^	1,184	0.60	0.20					0.79	0.69

a Number of compounds in the dataset;

b Tanimoto-distance threshold value;

c Smoothing factor;

d Pearson's correlation coefficient;

e *Regression model*.

#### Blood-brain barrier

The blood-brain barrier (BBB) is a highly selective barrier that separates the circulating blood from the central nervous system (CNS) (Abbott et al., 2006). It allows the passage of water molecules and water-soluble lipid molecules, as well as the selective transport of glucose and amino acids. The benefit of predicting BBB-permeable compounds is two-fold: (1) to identify toxicants that could harm the brain, and (2) to design drug molecules that can pass the BBB and reach their target in the CNS.

We developed a vNN-based BBB model, using 353 compounds whose BBB permeability values (log*BB*) were obtained from the literature (Muehlbacher et al., 2011; Naef, 2015). We classified compounds with log *BB* values of <−0.3 and >+0.3 as BBB non-permeable and permeable, respectively. To calculate performance measures, we classified BBB permeable and BBB non-permeable compounds as positives and negatives, respectively.

The model predicted whether or not a given compound would pass the BBB, but only for compounds within the applicability domain defined by the training set. The performance measures in Table 1 were calculated from 10-fold CV. The model showed a high overall accuracy of 90% and a kappa value of 0.80, with a coverage of 61%. The size of the dataset limited the applicability domain of the model. However, if new data become available, they can easily be added to the model to increase the applicability domain.

The model performed on par with the best of the BBB models published thus far. Most of the latter models, which used small datasets, are global models applied to any molecule. However, all models have a finite applicability domain (Cherkasov et al., 2014). Indeed, modeling BBB permeability is complicated because there are different possible routes across the barrier, via passive diffusion or protein transport, and no model singlehandedly accounts for all factors associated with this property. Our vNN model only makes predictions for compounds that are structurally similar enough to the test set molecules to ensure that they have the same type of transport mechanism. Thus, our vNN method accounts for multiple transport routes.

#### MMP disruption (mitochondrial toxicity)

Given the fundamental role of mitochondria in cellular energetics and oxidative stress, mitochondrial dysfunction has been implicated in cancer, diabetes, neurodegenerative disorders, and cardiovascular diseases (Pieczenik and Neustadt, 2007). Many pharmaceuticals and environmental toxicants cause mitochondrial dysfunction (Meyer et al., 2013). Therefore, the ability to predict the impact of chemicals on mitochondrial function would be useful. However, predicting mitochondrial toxicants is complicated because mitochondrial dysfunction can result from impairing any of the following: (1) the electron transport chain (ETC), (2) the mitochondrial transport pathway, (3) fatty acid oxidation, (4) the citric acid cycle, (5) mtDNA replication, (6) and mitochondrial protein synthesis.

There are several common experimental techniques to measure mitochondrial function. We used the largest dataset of chemical-induced changes in mitochondrial membrane potential (MMP), based on the assumption that a compound that causes mitochondrial dysfunction is also likely to reduce the MMP. We developed a vNN-based MMP prediction model, using 6,261 compounds collected from a previous study that screened a library of 10,000 compounds (~8,300 unique chemicals) at 15 concentrations, each in triplicate, to measure changes in the MMP in HepG2 cells (Attene-Ramos et al., 2015). The study found that 913 compounds decreased the MMP, whereas 5,395 compounds had no effect. We classified compounds that decreased the MMP as positives and those that did not affect the MMP as negatives.

Our MMP model predicted whether a given compound had the potential to affect the MMP and thereby cause mitochondrial dysfunction. It made predictions for compounds that were well represented in the applicability domain, but not for any other compound. The model showed a high overall accuracy of 89% and a kappa value of 0.61, with a coverage of 69% (Table 1).

#### Cytotoxicity (HepG2)

Cytotoxicity is the degree to which a chemical causes damage to cells. Cytotoxicity assays are widely used to screen compounds for unwanted cell damage, and to identify compounds that could be used, for example, to kill cancer cells. As such, the ability to identify cytotoxic compounds is highly desirable.

We developed a cytotoxicity prediction model, using a training dataset of *in vitro* toxicity against HepG2 cells for 6,097 structurally diverse compounds, which we collected from Chemical European Biology Laboratory (ChEMBL) (Bento et al., 2014). In developing our model, we considered compounds with an IC_50_ of 10 μM or less in the *in vitro* assay as cytotoxic. We classified cytotoxic compounds as positives and non-toxic compounds as negatives.

The cytotoxicity model performed well, with an overall accuracy of 84% and a kappa value of 0.64 (Table 1). Because compounds in the dataset achieved only sparse coverage of the chemical space, the model only predicted compounds that were well represented in the dataset. It did not give predictions for other compounds, and thereby avoided misleading results. When using 10-fold CV, the model reliably predicted 89% of the compounds in our dataset.

#### Drug-induced liver injury

Over the last 50 years, drug-induced liver injury (DILI) has been the most commonly cited reason for drug withdrawals from the market (Assis and Navarro, 2009). As a result, current drug development efforts are devoted to identifying and eliminating potential DILI compounds. Therefore, a model that predicts at an early stage whether a compound causes liver injury would be highly desirable. However, the mechanisms of DILI are complicated and diverse, making toxicology studies difficult. For example, compounds that cause DILI in humans do not necessarily induce clear liver injury in animal studies.

We collected DILI data from four sources used by Xu et al. (2015): (1) the U.S. FDA's National Center for Toxicological Research (NCTR dataset) (Chen M. et al., 2011), as well as the datasets of (2) Greene (Greene et al., 2010), (3) Xu (Xu et al., 2008), and (4) Liew (Liew et al., 2011). In the first three datasets, which included pharmaceuticals, we classified a compound as causing DILI if it was associated with a high risk of DILI and not if there was no such risk. We excluded low-risk DILI compounds. In the Liew dataset, which contained both pharmaceuticals and non-pharmaceuticals, we classified a compound as causing DILI if it was associated with any adverse liver effect. DILI-associated compounds were classified as positives and non-DILI compounds as negatives.

The performance measures of the vNN model, using 10-fold CV of the entire dataset excluding duplicated compounds, showed an overall accuracy of 71% and a coverage of 66% (Table 1). We also used the same datasets and compared our models with some previously published deep learning models (Xu et al., 2015; Table 2). Considering the complexity and computational time investment involved in training these deep learning models, our vNN models performed relatively well; they performed on-par with the deep learning models, albeit with a coverage ranging from 40 to 65%.

**Table 2:** Performance measures of vNN DILI models compared with deep learning.

	**NCTR^a^**	**NCTR^a^**	**Green^a^**	**Xu^a^**	**Combined^a^**
	**10-fold CV**	**Test**	**Test**	**Test**	**10-fold CV**
Accuracy (%)	87 (81)	75 (70)	61 (65)	60 (62)	85 (85)^b^
Sensitivity (%)	65 (70)	64 (80)	51 (75)	52 (62)	83 (84)^b^
Specificity (%)	95 (88)	86 (60)	75 (46)	70 (62)	88 (85)^b^
Coverage (%)	40	50	46	41	67
No of Compounds	190	185	320	236	475

a *Values in parentheses are the deep learning results from Xu et al. (2015)*.

b *Values averaged over 60 runs of 10-fold CV*.

#### Cytochrome P450 inhibition (drug-drug interaction)

Cytochrome P450 enzymes (CYPs) constitute a superfamily of proteins that play an important role in the metabolism and detoxification of xenobiotics (Brown et al., 2008). A drug should not be rapidly metabolized by CYPs if it is to maintain an effective concentration. In addition, it should not inhibit drug-metabolizing CYPs, because such an effect could elevate the concentration of a co-administered drug and potentially lead to drug overdose—an effect known as a drug-drug interaction (Murray, 2006). In drug development, *in vitro* assays are routinely used to assess interactions between drug candidates and CYPs. However, there is a need for *in silico* models that assess potential interactions with CYPs in the early stages of drug development.

We collected data for five main drug-metabolizing CYPs: 1A2, 2D6, 2C9, 2C19, and 3A4. We retrieved CYP inhibitors from ChEMBL (Bento et al., 2014) and classified them as inhibitors if the IC_50_ was below 10 μM. We removed from the dataset any duplicates or compounds tested multiple times with contradicting results, in which the reported IC_50_ values were below and above the 10 μM threshold value. For all CYPs, we classified inhibitors and non-inhibitors as positives and negatives, respectively.

The performance measures for the five CYP models are presented in Table 1. All models achieved high accuracy (87–91%) and kappa values (0.54–0.68) while maintaining high coverage (75–78%).

#### hERG blockers

The human ether-à-go-go-related gene (hERG) codes for a potassium ion channel involved in the normal cardiac repolarization activity of the heart (Sanguinetti and Tristani-Firouzi, 2006). Drug-induced blockade of hERG function can cause long QT syndrome, which may result in arrhythmia and death (De Ponti et al., 2001). For this reason, hERG liability is one of the toxicology screens that drug candidates must pass during early pre-clinical studies. Therefore, *in silico* models that identify hERG blockers in the early stages of drug design are of considerable interest.

We retrieved 282 known hERG blockers from the literature and classified compounds with an IC_50_ cutoff value of 10 μM or less as blockers (Wang et al., 2012). We also collected a set of 404 compounds with IC_50_ values >10 μM from ChEMBL (Bento et al., 2014) and classified them as non-blockers (Czodrowski, 2013). We classified hERG blockers and non-blockers as positives and negatives, respectively.

The hERG model performed with an overall accuracy of 84%, well-balanced sensitivity and specificity values (84 and 83%, respectively), and a kappa value of 0.68 (Table 1). The model reliably predicted 80% of the compounds in our dataset when using 10-fold CV. However, the coverage of chemical space by the non-hERG blockers in the dataset was sparse, and only compounds well represented in the dataset were predicted with confidence. Because the model did not give predictions for other compounds, it avoided misleading results. Therefore, users should use this model to flag potential hERG blockers rather than to identify non-hERG blockers.

#### Pgp substrates and inhibitors

P-glycoprotein (Pgp) is an essential cell membrane protein that extracts many foreign substances from the cell (Ambudkar et al., 2003). As such, it is a critical determinant of the pharmacokinetic properties of drugs. Cancer cells often overexpress Pgp, which increases the efflux of chemotherapeutic agents from the cell and prevents treatment by reducing the effective intracellular concentrations of such agents—a phenomenon known as multidrug resistance (Borst and Elferink, 2002). For this reason, identifying compounds that can either be transported out of the cell by Pgp (substrates) or impair Pgp function (inhibitors) is of great interest. Therefore, using the vNN method, we developed models to predict both Pgp substrates and Pgp inhibitors.

The Pgp substrate dataset was collected by Hou and co-workers (Li et al., 2014). This dataset included measurements for 422 substrates and 400 non-substrates. To generate a large Pgp inhibitor dataset, we combined two datasets (Broccatelli et al., 2011; Chen L. et al., 2011), and removed duplicates to form a combined dataset consisting of a training set of 1,319 inhibitors and 937 non-inhibitors. We classified the Pgp inhibitors (substrates) and non-inhibitors (non-substrates) as positives and negatives, respectively.

The vNN models for identifying Pgp substrates and inhibitors gave accurate and reliable results, showing overall accuracies of 79 and 85%, respectively, when using 10-fold CV, with corresponding kappa values of 0.58 and 0.66. These models reliably predicted 65 and 76% of the compounds in their datasets to be Pgp substrates and inhibitors, respectively. The performance characteristics of these models were comparable, or at times superior, to those of other model constructs (Schyman et al., 2016).

#### Chemical mutagenicity (AMES test)

Mutagens are chemicals that cause abnormal genetic mutations leading to cancer. A common way to assess a chemical's mutagenicity is the Ames test (Ames et al., 1973). This test has become the standard for assessing the safety of chemicals and drugs, and has been used to test thousands of molecules. We examined whether the vNN method could effectively use existing data to predict mutagenicity.

We retrieved an Ames mutagenicity dataset consisting of 6,512 compounds, of which 3,503 were Ames-positive (Hansen et al., 2009), and developed a vNN Ames mutagenicity prediction model. The model performed well, with an overall accuracy of 82%; sensitivity and specificity values of 86 and 75%, respectively; and a high kappa value of 0.62 (Table 1). The model also reliably predicted 79% of the compounds in the Ames dataset when using 10-fold CV. Further details of the model and its prediction performance can be found elsewhere (Liu and Wallqvist, 2014).

#### Maximum recommended therapeutic dose

A basic principle of toxicology is that “the dose makes the poison.” For most drugs, the therapeutic dose is limited by toxicity, and the maximum recommended therapeutic dose (MRTD) is an estimated upper daily dose that is safe (Contrera et al., 2004). Investigators carry out toxicological experiments on animals to determine the toxic effects of a drug and the initial dose for human clinical trials. Unfortunately, there is a lack of correlation between animal and human toxicity data. Therefore, we investigated whether the vNN method could predict the MRTD values of new compounds based on known human MRTD data. If so, the values could be used to estimate the starting dose in phase I clinical trials, while significantly reducing the number of animals used in preliminary toxicology studies.

We obtained a dataset of MRTD values publically disclosed by the FDA, mostly of single-day oral doses for an average adult with a body weight of 60 kg, for 1,220 compounds (most of which are small organic drugs). For modeling purposes we converted the MRTD unit from mg/kg-body weight/day to mol/kg-body weight/day via the molecular weight of the compound. However, the predicted values on the website are reported in mg/day based upon an average adult weighing 60 kg. We excluded organometallics, high-molecular weight polymers (>5,000 Da), nonorganic chemicals, mixtures of chemicals, and very small molecules (<100 Da). We used an external test set of 160 compounds, which was collected by the FDA for validation. The total dataset for our model contained 1,184 compounds (Liu et al., 2012).

The MRTD model reliably predicted 69% of the FDA MRTD dataset, with a Pearson's correlation coefficient (*R*) of 0.79 between the predicted and measured *log*(MRTD) values, and a mean deviation (mDev) of 0.56 *log* units, using 40-fold CV (Liu et al., 2012). For comparison, we used two popular QSAR regression methods—the partial least square (PLS) and support vector machine (SVM) methods—to develop two global models to fit the training dataset. We evaluated the model performance, using 40-fold CV of the training set. The best PLS model achieved an *R*-value of 0.50 and an mDev of 0.79. The results for the SVM model were at best comparable to those of the best PLS model, with an *R*-value of 0.53 and an mDev of 0.63. For further details of the model, we refer the reader to our previous paper (Liu et al., 2012).

#### Human liver microsomal stability

The human liver is the most important organ for drug metabolism. For a drug to achieve effective therapeutic concentrations in the body, it cannot be metabolized too rapidly by the liver. Otherwise, it would need to be administered at high doses, which are associated with high toxicity. To identify and exclude rapidly metabolized compounds (Di et al., 2003), pharmaceutical companies commonly use the human liver microsomal (HLM) stability assay. This has led to the accumulation of a substantial body of HLM stability data in publicly accessible databases.

However, our knowledge of how enzymes in the HLM assay metabolize drugs remains fragmentary. Therefore, we examined whether the vNN method could effectively predict drugs that are rapidly metabolized by the liver. We retrieved HLM data from the ChEMBL database (Bento et al., 2014), manually curated the data, and classified compounds as stable or unstable based on the reported half-life [T1/2 > 30 min was considered stable, and T1/2 < 30 min unstable (Liu et al., 2015)]. The final dataset contained 3,219 compounds. Of these, we classified 2,047 as stable and 1,166 as unstable.

The HLM model performed with an overall accuracy of 81%; sensitivity and specificity values of 71 and 87%, respectively; and a high kappa value of 0.60 (Table 1). The HLM model reliably predicted 91% of the compounds in the HLM dataset when using 10-fold CV. We refer the reader to our original paper for further details of the model and its prediction performance (Liu et al., 2015).

### Implementation aspects

The vNN-ADMET web-application is hosted on an Apache Tomcat Web server that is accessible via a secure service over Hypertext Transfer Protocol Secure (https). We developed the application on the basis of a three-tiered architecture, composed of a backend database, controller, and presentation tiers. The first tier consists of a PostgreSQL 9.5.7 database that stores user account information, uploaded files, constructed models, and model predictions. The second (controller) tier provides access to the prediction engine and implements the functionality required to create and manage multiple predictions. We implemented this tier, using Pipeline Pilot protocols hosted on a local Pipeline Pilot server. The third (presentation) tier provides for visualization of the results, with plotting capabilities for multiple predictions. The controller and presentation tiers were developed using Java Platform, Enterprise Edition 7, Spring Framework 4.2.2, JavaServer Faces 2.2, PrimeFaces 6.0, and BootsFaces 1.0.2. The graphical user interface in the presentation tier uses Web standards supported by modern Web browsers, including Microsoft Edge 38, Chrome version 58, and Firefox version 53, without any need for plugins.

To use the system, the user must register for an account at https://vnnadmet.bhsai.org/. Once logged in, the user can build custom models, and run pre-built ADMET and custom models. The data corresponding to a user (login credentials, compounds, models, results, etc.) are not shared with any other user within or outside the system. The uploaded compounds, constructed models, and model predictions are purged from the system every 2 weeks.

## Discussion

We have presented a web-based vNN prediction platform, with which a user can build and test models as well as predict the ADMET properties of a compound by using our existing tools.

All vNN models performed well with accuracies of >71% (see Table 1 for further details). On average, the models predicted 75% of the compounds in their datasets, using 10-fold CV.

Achieving fair comparisons between a new model and a competing model is always difficult because such comparisons require the same training data, validation data, and performance measures. An important advantage of our platform is that it offers an opportunity for developers to compare their methods with our vNN method, using their training and validation data.

For demonstrative purposes, we quantitatively compared our vNN method with the winning method of the Tox21 challenge (Huang et al., 2016). This challenge was issued in 2014 by the U.S. Toxicology in the twenty-first Century (Tox21) program, which aims to improve toxicity prediction methods. The Tox 21 consortium solicited models that could best predict the toxicity of 10,000 compounds it had tested in 12 different assays (Table 3). It used a final evaluation dataset that was concealed to determine the winners.

**Table 3:** Tox21 assays with PubChem assay identification number.

**Assay ID**	**Assay**	**PubChem AID**
AhR	Aryl hydrocarbon receptor	743122
Aromatase	Aromatase	743139
AR	Androgen receptor	743040
AR-LBD	Androgen receptor LBD	743053
ER	Estrogen receptor alpha	743079
ER-LBD	Estrogen receptor alpha LBD	743077
PPAR-g	Peroxisome proliferator-activated receptor gamma	743140
ARE	Nuclear factor antioxidant responsive element	743219
ATAD5	ATAD5	720516
HSE	Heat shock factor response element	743228
MMP	Mitochondrial membrane potential	720637
p53	p53	720552

Table 4 shows the area under the curve for the receiver operating characteristic (AUC-ROC) of the 18 leading research teams with their best-performing model for each of the 12 assays. To compare our models with those in Table 4, we set *d* to 1.0 so that we could predict all compounds. The vNN method performed reasonably well in predicting most of the Tox21 assays. We note that the grand challenge winner used data from PubChem (Wang et al., 2009) and ChEMBL (Bento et al., 2014), in addition to the Tox21 data, which makes it impossible for us to directly compare our results with their results.

**Table 4:** AUC-ROCs of vNN models and the best 18 models on the final evaluation test of the Tox21 Challenge.

**Team**	**AhR**	**AR**	**AR-LBD**	**ARE**	**Aromatase**	**ATAD5**	**ER**	**ER-LBD**	**HSE**	**MMP**	**p53**	**PPARg**
GrandWinner	0.928	0.807	0.879	0.840	0.834	0.793	0.810	0.814	0.865	0.942	0.862	0.861
AMAZIZ	0.913	0.770	0.846	0.805	0.819	0.828	0.806	0.806	0.842	0.95	0.843	0.830
dmlab	0.781	0.828	0.819	0.768	0.838	0.800	0.766	0.772	0.855	0.946	0.880	0.831
T	0.913	0.676	0.848	0.801	0.825	0.814	0.784	0.805	0.811	0.937	0.847	0.822
Microsomes	0.901	–	–	0.804	–	0.812	0.785	0.827	–	–	0.826	0.717
FilipsPL	0.893	0.736	0.743	0.758	0.776	–	0.771	–	0.766	0.928	0.815	–
Charite	0.896	0.688	0.789	0.739	0.781	0.751	0.707	0.798	0.852	0.880	0.834	0.7
RCC	0.872	0.763	0.747	0.761	0.792	0.673	0.781	0.762	0.755	0.920	0.795	0.637
Frozenarm	0.865	0.744	0.722	0.700	0.740	0.726	0.745	0.790	0.752	0.859	0.803	0.803
ToxFit	0.862	0.744	0.757	0.697	0.738	0.729	0.729	0.752	0.689	0.862	0.803	0.791
CGL	0.866	0.742	0.566	0.747	0.749	0.737	0.759	0.727	0.775	0.880	0.817	0.738
SuperToX	0.854	–	0.560	0.711	0.742	–	–	–	–	0.862	0.732	–
Kibutz	0.865	0.750	0.694	0.708	0.729	0.737	0.757	0.779	0.587	0.838	0.787	0.666
MML	0.871	0.693	0.660	0.701	0.709	0.749	0.750	0.710	0.647	0.854	0.815	0.645
NCI	0.812	0.628	0.592	0.783	0.698	0.714	0.483	0.703	0.858	0.851	0.747	0.736
VIF	0.827	0.797	0.610	0.636	0.671	0.656	0.732	0.735	0.723	0.796	0.648	0.666
Toxic Avg	0.715	0.721	0.611	0.633	0.671	0.593	0.646	0.640	0.465	0.732	0.614	0.682
Swamidass	0.353	0.571	0.748	0.372	0.274	0.391	0.680	0.738	0.711	0.828	0.661	0.585
vNN	0.883	0.716	0.626	0.727	0.786	0.699	0.738	0.770	0.793	0.882	0.808	0.690
vNN rank	7	12	13	11	6	13	12	9	7	7	10	11

*The vNN parameters were set to h = 0.3 and d_0_ = 1.0. Gray cells indicate models showing performance inferior to the vNN models*.

The MMP data we used for our mitochondrial dysfunction model were the same as those used in the Tox21 challenge (Attene-Ramos et al., 2015; Huang et al., 2016). Our MMP model was the seventh best performing model, with an AUC-ROC value of 0.882 (with *h* = 0.3 and *d* = 1.0). This was comparable to the values of more elaborate and computationally time-consuming methods, such as deep learning (Table 4).

Some QSAR methods do not use an applicability domain to determine whether their predictions are reliable. This could lead to the misperception that a model can predict the activity of any molecule. The applicability domain is vital to the vNN method. The user of our platform can adjust it by varying the Tanimoto distance threshold value. Although this could be set to 1 so that the model predicts the activity of any molecule, no model is likely to have an unlimited applicability domain (Liu et al., 2015).

A more reasonable approach to improve a vNN-based model is to increase the applicability domain by adding more reference compounds. A good test of the power of a model to generate prospective predictions is time-split validation, which divides the data into “old” and “new” data and uses the former to train the model and the latter “new” data for validation (Sheridan, 2013; Liu et al., 2015). We have previously shown in a time-split validation that, whereas the accuracy of a vNN model is roughly maintained, the number of “new” compounds that it can predict is significantly reduced. However, by simply adding a few “new” compounds, the coverage increases significantly (Liu et al., 2015).

The lack of training data poses an important limitation to the vNN approach. When a dataset is too small, there is a high probability that a target molecule will have no qualified near neighbors in the dataset, and hence a high-quality prediction cannot be made. However, the lack of training data is a limitation for all machine learning methods. The difference is that most such methods build a model no matter how small the training dataset, and will always make a prediction for any input molecule without considering the reliability of the predicted result. In our view, it is better not to give a prediction at all if it is unreliable. This also alerts users to use alternative methods, including experimental measurements, to derive a reliable answer. As more experimental data become available over time, the performance of the vNN method will improve without retraining. This is in contrast to most other machine learning methods, which cannot take advantage of new data without retraining a model.

This finding is especially significant for drug discovery labs because the chemical space is restricted by the target candidates they are investigating. For example, when exploring a new drug target, it is crucial to continuously update the model with new data to ensure that the applicability domain is relevant for the new target. In a vNN-based model, this can be done easily by adding the SMILES strings of the new compounds to the reference dataset. For this reason, we believe that our web-based vNN platform has the potential to greatly accelerate the development of drugs.

## Author contributions

PS, RL, and AW developed the method, analyzed the data, and wrote the manuscript. VD designed and implemented the web server.

### Conflict of interest statement

The authors declare that the research was conducted in the absence of any commercial or financial relationships that could be construed as a potential conflict of interest.

## Abbreviations

Pgp: permeability glycoprotein
MDR: multidrug resistance.
