# Supplementary material for: vNN Web Server for ADMET Predictions
Source: Front Pharmacol. 2017 Dec 4;8:889. doi: 10.3389/fphar.2017.00889 (PMC5722789; doi:10.3389/fphar.2017.00889)
Supplement: Supplementary file 1 [file Table1.DOCX]

Supplementary Material

vNN Web Server for ADMET Predictions

Patric Schyman*, Ruifeng Liu, Valmik Desai and Anders Wallqvist*

DoD Biotechnology High Performance Computing Software Applications Institute, Telemedicine and Advanced Technology Research Center, U.S. Army Medical Research and Materiel Command, Fort Detrick, MD, USA

*** Correspondence:**Anders Wallqvist

E-mail [sven.a.wallqvist.civ@mail.mil](mailto:sven.a.wallqvist.civ@mail.mil)

Patric Schyman

E-mail: [pschyman@bhsai.org](mailto:pschyman@bhsai.org)

**Table S1. Performance measures of vNN models in 10-fold cross validation using a restricted or unrestricted applicability domain.**

| Model | Data^a^ | $d_{0}$^b^ | $h$^c^ | Accuracy | Sensitivity | Specificity | kappa | R^d^ | Coverage |
| --- | --- | --- | --- | --- | --- | --- | --- | --- | --- |
| DILI | 1427 | 0.60 | 0.50 | 0.71 | 0.70 | 0.73 | 0.42 |  | 0.66 |
|  |  | 1.00 | 0.20 | 0.67 | 0.62 | 0.72 | 0.34 |  | 1.00 |
| Cytotox (hep2g) | 6097 | 0.40 | 0.20 | 0.84 | 0.88 | 0.76 | 0.64 |  | 0.89 |
|  |  | 1.00 | 0.20 | 0.84 | 0.73 | 0.89 | 0.62 |  | 1.00 |
| HLM | 3219 | 0.40 | 0.20 | 0.81 | 0.72 | 0.87 | 0.59 |  | 0.91 |
|  |  | 1.00 | 0.20 | 0.81 | 0.70 | 0.87 | 0.57 |  | 1.00 |
| CYP 1A2 | 7558 | 0.50 | 0.20 | 0.90 | 0.70 | 0.95 | 0.66 |  | 0.75 |
|  |  | 1.00 | 0.20 | 0.89 | 0.61 | 0.95 | 0.60 |  | 1.00 |
| CYP 2C9 | 8072 | 0.50 | 0.20 | 0.91 | 0.55 | 0.96 | 0.54 |  | 0.76 |
|  |  | 1.00 | 0.20 | 0.90 | 0.44 | 0.96 | 0.46 |  | 1.00 |
| CYP 2C19 | 8155 | 0.50 | 0.20 | 0.87 | 0.64 | 0.93 | 0.58 |  | 0.76 |
|  |  | 1.00 | 0.20 | 0.86 | 0.52 | 0.94 | 0.50 |  | 1.00 |
| CYP 3A4 | 10373 | 0.50 | 0.20 | 0.88 | 0.76 | 0.92 | 0.68 |  | 0.78 |
|  |  | 1.00 | 0.20 | 0.88 | 0.69 | 0.93 | 0.64 |  | 1.00 |
| CYP 2D6 | 7805 | 0.50 | 0.20 | 0.89 | 0.61 | 0.94 | 0.57 |  | 0.75 |
|  |  | 1.00 | 0.20 | 0.88 | 0.52 | 0.95 | 0.51 |  | 1.00 |
| BBB | 353 | 0.60 | 0.20 | 0.90 | 0.94 | 0.86 | 0.80 |  | 0.61 |
|  |  | 1.00 | 0.10 | 0.82 | 0.88 | 0.75 | 0.64 |  | 1.00 |
| Pgp Substrate | 822 | 0.60 | 0.20 | 0.79 | 0.80 | 0.79 | 0.58 |  | 0.66 |
|  |  | 1.00 | 0.20 | 0.73 | 0.73 | 0.74 | 0.47 |  | 1.00 |
| Pgp Inhibitor | 2304 | 0.50 | 0.20 | 0.85 | 0.91 | 0.73 | 0.66 |  | 0.76 |
|  |  | 1.00 | 0.10 | 0.81 | 0.86 | 0.74 | 0.61 |  | 1.00 |
| hERG | 685 | 0.70 | 0.70 | 0.84 | 0.84 | 0.83 | 0.68 |  | 0.80 |
|  |  | 1.00 | 0.20 | 0.82 | 0.82 | 0.83 | 0.64 |  | 1.00 |
| MMP | 6261 | 0.50 | 0.40 | 0.89 | 0.64 | 0.94 | 0.61 |  | 0.69 |
|  |  | 1.00 | 0.20 | 0.87 | 0.52 | 0.94 | 0.50 |  | 1.00 |
| AMES | 6512 | 0.50 | 0.40 | 0.82 | 0.86 | 0.75 | 0.62 |  | 0.79 |
|  |  | 1.00 | 0.20 | 0.79 | 0.82 | 0.75 | 0.57 |  | 1.00 |
| MRTD^e^ | 1184 | 0.6 | 0.2 |  |  |  |  | 0.79 | 0.69 |
|  |  | 1.00 | 0.20 |  |  |  |  | 0.74 | 1.00 |

^a^Number of compounds in the dataset; ^b^Tanimoto-distance threshold value; ^c^Smoothing factor; ^d^Pearson’s correlation coefficient ; ^e^Regression model.
